# Acute Exercise and Motor Memory Consolidation: The Role of Exercise Intensity

**DOI:** 10.1371/journal.pone.0159589

**Published:** 2016-07-25

**Authors:** Richard Thomas, Line K. Johnsen, Svend S. Geertsen, Lasse Christiansen, Christian Ritz, Marc Roig, Jesper Lundbye-Jensen

**Affiliations:** 1 Department of Nutrition, Exercise and Sports, University of Copenhagen, Copenhagen, Denmark; 2 Department of Neuroscience & Pharmacology, University of Copenhagen, Copenhagen, Denmark; 3 Department of Neurological Surgery, The Miami Project to Cure Paralysis, University of Miami, Miami, Florida, United States of America; 4 School of Physical & Occupational Therapy, Faculty of Medicine, McGill University, Montréal, Québec, Canada; 5 Memory and Motor Rehabilitation Laboratory (MEMORY-LAB), Jewish Rehabilitation Hospital, Montréal Center for Interdisciplinary Research in Rehabilitation (CRIR), Montréal, Québec, Canada; University of Birmingham, UNITED KINGDOM

## Abstract

A single bout of high intensity aerobic exercise (~90% VO_2peak_) was previously demonstrated to amplify off-line gains in skill level during the consolidation phase of procedural memory. High intensity exercise is not always a viable option for many patient groups or in a rehabilitation setting where low to moderate intensities may be more suitable. The aim of this study was to investigate the role of intensity in mediating the effects of acute cardiovascular exercise on motor skill learning. We investigated the effects of different exercise intensities on the retention (performance score) of a visuomotor accuracy tracking task. Thirty six healthy male subjects were randomly assigned to one of three groups that performed either a single bout of aerobic exercise at 20 min post motor skill learning at 45% (EX45), 90% (EX90) maximal power output (W_max_) or rested (CON). Randomization was stratified to ensure that the groups were matched for relative peak oxygen consumption (ml O_2_/min/kg) and baseline score in the tracking task. Retention tests were carried out at 1 (R1) and 7 days (R7) post motor skill learning. At R1, changes in performance scores were greater for EX90 compared to CON (p<0.001) and EX45 (p = 0.011). The EX45 and EX90 groups demonstrated a greater change in performance score at R7 compared to the CON group (p = 0.003 and p<0.001, respectively). The change in performance score for EX90 at R7 was also greater than EX45 (p = 0.049). We suggest that exercise intensity plays an important role in modulating the effects that a single bout of cardiovascular exercise has on the consolidation phase following motor skill learning. There appears to be a dose-response relationship in favour of higher intensity exercise in order to augment off-line effects and strengthen procedural memory.

## Introduction

The benefits of physical activity on brain function, brain health and cognition are well documented [1–8] and it has previously been demonstrated that an acute bout of exercise can positively affect cognition [9, 10] and declarative memory [11, 12]. The relationship between exercise and memory has been summarized in a meta-analysis by Roig and colleagues and the evidence points towards acute exercise having positive effects on long-term memory. Results vary greatly however, depending on the type of task and memory studied [13]. Recent studies have reported that an acute bout of high intensity aerobic exercise can also improve motor skill learning [14–16]. While the different phases of the memory formation process; acquisition, consolidation and retrieval/retention encompass several neural processes and involve a vast number of interconnected brain networks [17–19], these studies provide evidence supporting the idea that an acute bout of cardiovascular exercise can positively affect procedural memory by affecting underlying cellular mechanisms in the period following skill practice (off-line effects).

While there is evidence to suggest that an acute exercise bout can positively affect procedural memory, the mechanisms underlying the effects remain, however, poorly understood and learning tasks and exercise paradigms vary between studies [14, 20–22]. It is thus, not clear which aspects of exercise are critical for positive effects on procedural memory. Acute exercise interventions do, however, allow for readily controlling and monitoring specific parameters (intensity, duration, modality) when investigating effects on procedural memory. Specifically, within exercise, it is possible to control intensity; either relative to maximal oxygen uptake, maximum power output or age related maximum heart rate.

In relation to procedural memory, Statton and colleagues [20] recently investigated the effect of an acute bout of moderate intensity exercise (30 min running at 65–85% of age predicted maximum heart rate) on motor skill acquisition and retention with the Sequential Visual Isometric Pinch Task (SVIPT). When moderate intensity exercise was performed prior to motor practice, exercise improved acquisition. There was however no effect of exercise on retention level over multiple days of training. The finding that moderate intensity exercise prior to motor practice influence skill acquisition but not delayed retention was also recently found by Snow and co-workers [21].

Recent work by Roig and colleagues [14] investigated the effects of an acute high intensity (90% VO_2peak_) exercise bout (cycling) on motor skill learning. The results showed that a bout of high intensity exercise performed prior to motor skill acquisition had a significant positive effect not on skill acquisition but on procedural memory assessed with delayed retention tests. Mang and co-workers also observed improvements in sequence-specific implicit motor learning at a 24h retention test when an acute bout of high intensity exercise, similar to the protocol from the study by Roig et al., was performed prior to acquisition [15]. Importantly, Roig and colleagues also found positive effects of intense exercise *following* motor practice, and in this case, the intense exercise bout had an even greater effect on consolidation of the procedural memory compared to when performed prior to motor practice [14]. The finding that exercise following motor practice can promote retention of procedural memory was recently confirmed by Thomas et al. [23].

These studies add credence to the hypothesis that exercise intensity (and timing) is somehow intimately related to improvements in the acquisition and retention of motor skills. Whereas moderate intensity exercise prior to motor practice had beneficial effects on acquisition, this was not found for high intensity exercise. High intensity exercise prior to and following motor practice had positive effects on consolidation of procedural memory and delayed retention. While these positive effects of intense exercise were found in delayed retention tests, the importance of exercise intensity following encoding or acquisition is however currently unclear, since only effects of high intensity exercise have been elucidated.

As exercise intensity is increased circulating concentrations of catecholamines, serotonin (5-HT), lactate, dopamine, insulin-like growth factor 1 (IGF-1) and vascular endothelial growth factor (VEGF) and Brain Derived Neurotrophic Factor (BDNF) increase concurrently [11, 16]. Different measures of corticospinal excitability (CSE) have also been reported to be affected by an acute bout of aerobic exercise [24, 25]. The specific role of these biomarkers and changes in CSE in relation to procedural memory are currently also poorly understood, but they are potential candidates influencing the process. Furthermore, their direct relationship to exercise intensity means that their potential contribution should be carefully considered.

High intensity exercise bouts may be too challenging and not appropriate for certain patient groups or in a rehabilitation setting. We planned to investigate the role of exercise intensity on the consolidation of motor skill learning via a visuomotor tracking task. We, therefore, aimed to investigate to what extent a low to moderate aerobic exercise bout of a similar duration affects motor skill learning when performed post acquisition and measured with delayed retention tests. We proposed the following hypothesis; the level of consolidation of a newly acquired motor skill, measured via retention tests at 1 (R1) and 7 days (R7), would depend on the intensity of the exercise performed in a dose-response manner. The higher the intensity the more pronounced the consolidation of the long term memory.

## Materials & Methods

### Subjects

Thirty-six able-bodied, healthy, right-handed males, 24 ± 3 (SD) years old were recruited from the Copenhagen area to participate in the study (Table 1). Right-handedness for each subject was evaluated with the Edinburgh Handedness Inventory (84.4 ± 21.7) [26]. At the time of recruitment for the study all subjects were naïve to the VAT used to investigate motor skill learning and procedural memory. Exclusion criteria for participation in the study included: age below 20 or over 35, body mass index (BMI) above 30, a history of neurological, psychiatric or medical diseases and a current intake of medication and/or recreational drugs, which could have an impact on learning and/or the central nervous system. All subjects gave their written informed consent prior to testing. The experiments were approved by the local ethics committee for the Greater Copenhagen area (protocol H-2-2011-032) and the study was performed in accordance with the declaration of Helsinki.

**Table 1:** Descriptive data of study participants (group mean ± SD).

	CON	EX45	EX90
No. of subjects	12	12	12
Age (years)	24.2 ± 3.0	23.5 ± 2.3	24.3 ± 2.3
Weight (kg)	81.7 ± 10.0	80.4 ± 6.5	77.9 ± 12.5
Height (cm)	185.8 ± 6.0	186.5 ± 6.8	180.1 ± 9.1
BMI (kg/m^2^)	23.7 ± 2.6	23.4 ± 1.0	23.9 ± 2.4
IPAQ (low/moderate/high)	0/4/8	0/2/10	0/2/10
VO_2peak_ (ml O_2_/min/kg)	51.0 ± 4.6	49.7 ± 3.8	51.1 ± 4.6
W_max_ (W)	325.0 ± 50.0	325.0 ± 39.9	320.8 ± 39.7
Baseline VAT Score	51.5 ± 8.9	52.4 ± 9.6	49.2 ± 9.3

BMI = Body Mass Index, IPAQ = International Physical Activity Questionnaire (Long)

### Visuomotor Accuracy Tracking Task (VAT)

**Fig 1:**
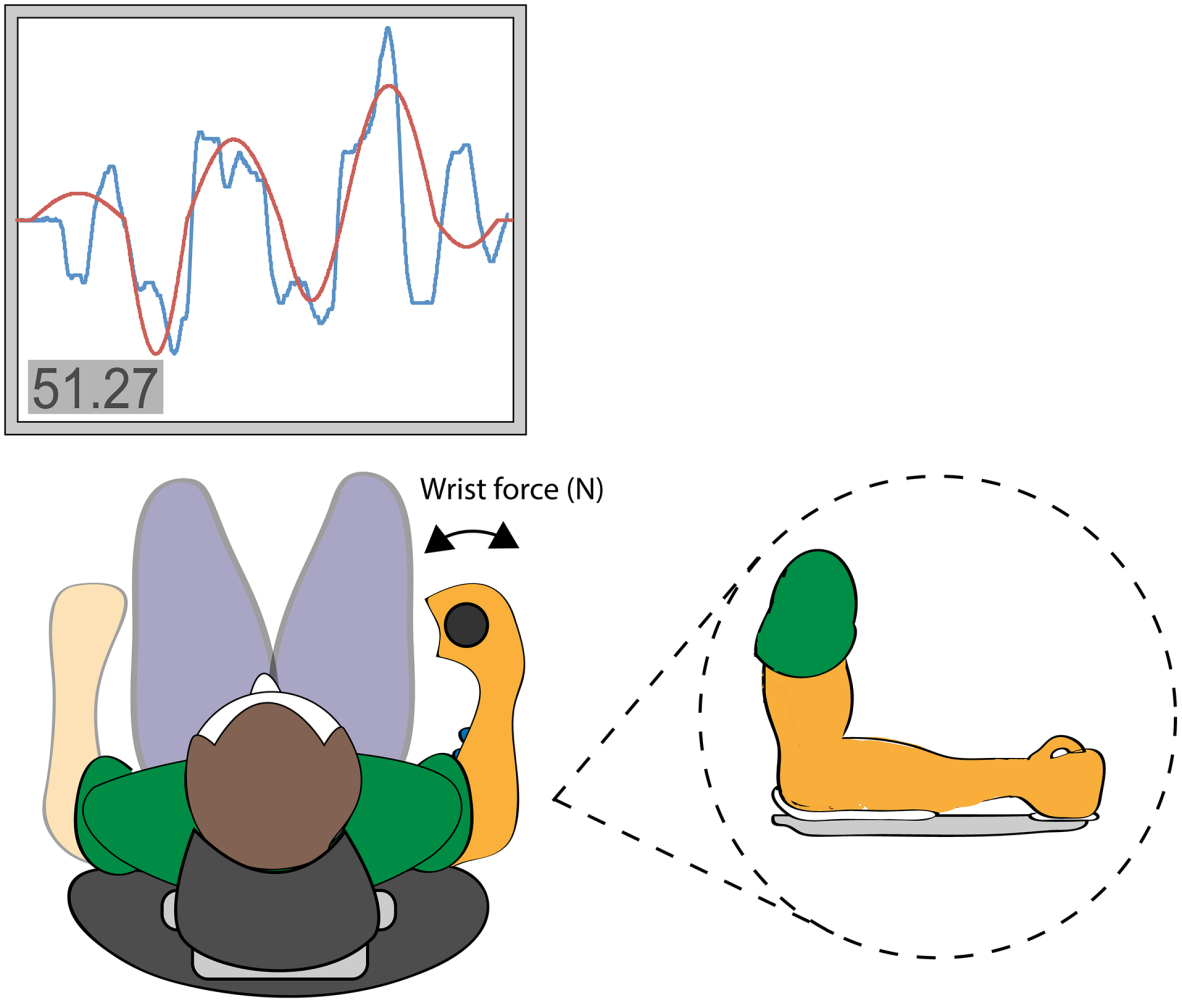
The visuomotor accuracy tracking task (VAT). Illustration of the setup for the visuomotor accuracy tracking task (VAT).

Subjects were seated on a 65cm high chair at a table in front of a computer screen. Their right forearm was fixed in a custom made frame with the elbow joint angle at 100–110° while their left arm rested on the table in front of them. At the distal end of the custom made frame a vertical cylindrical handle with a diameter of 2.5cm and a length of 14.5cm allowed subjects to grip with a completely closed fist. The handle was attached to a strain gauge, which transferred information on the torque created. Subjects were able to apply force to the handle with isometric muscle contractions in a lateral and medial direction (i.e. wrist extension and flexion). See Fig 1 for an illustration of the VAT setup.

Each VAT trial consisted of a fixed target consisting of a triple sine wave curve (see Fig 1), which subjects then had to track as accurately as possible by moving a cursor trace up and down respectively with wrist extension moving the cursor upwards and flexion moving it downwards. With no lateral or medial force applied to the handle the cursor would return to the pre-determined baseline position. The cursor moved with a constant velocity from left to right taking 8s for each trial. The closer the cursor was moved to the target, the better the score.

Handle torque force was digitized and sampled at 500Hz with a USB 6228 DAQ (Data Acquisition) board (National Instruments Inc., USA). A customized script built on MATLAB (MathWorks Inc., USA) was used to run the VAT. After each trial feedback on performance was provided on the screen by means of a score from 0–100. The score was calculated as a reverse function of the measure of total error between the double sine curve and the trace produced by the subject. The error score was defined as the mean vertical distance from the fixed target in relation to its distance from the “zero” position of the cursor when force = 0N. If the root mean square (RMS) error exceeded two times the distance from target to baseline the score was zero. A RMS error of 0 equaled a score of 100. This was calculated at all sampled data points across each trial. Subjects performed the VAT on three occasions: at the main experiment (acquisition), at the 1 day retention test (R1) and at the 7 day retention test (R7). The acquisition phase consisted of 5 blocks (B1, B2, B3, B4, B5) of 20 trials (100 trials in total) with each block taking 4 min to complete with rest periods of 2 min between blocks giving a total time of 28 min performing the VAT.

Before starting the VAT at acquisition, R1 and R7 all subjects performed a familiarization trial with a single sine wave and had to score ≥50 three times consecutively with the correct tracking direction at the first deflection of the target in order to proceed. This was done to minimize differences at baseline that could have affected the final levels of performance at block 5 (B5). At the start of all VAT sessions the apparatus was reset with the subject holding the handle at rest with their wrist in the neutral start position. Furthermore, at acquisition, the VAT was introduced via a standardized presentation lasting 5 minutes. The VAT model was based on similar visuomotor tasks used to induce a robust learning effect [14, 27–29].

Baseline was defined as the mean score for trials 2–20 in block 1 (B1) and Block 5 (B5) represented the post motor learning level. A single block of 20 trials was performed at R1 and R7 without feedback (score) for each trial [30]. The mean score for trial 2–20 in each block was used as a measure of retention. The first trial was omitted to ensure any adverse effects on the retention tests due to large variability relating to initiation of the test block. The 7 day retention test (R7) included an additional training block, R7Tr, consisting of 20 trials with feedback as the performance score. This was used to check for saturation in the VAT and to assess for a potential ceiling effect in skill level where mean scores (trial 2–20) were compared to R7. Subjects therefore performed a total of 160 trials throughout the whole experiment.

### Graded Maximal Exercise Test

The graded maximal exercise test was conducted in order to assess the subject’s aerobic fitness level (VO_2peak_), maximal power output (W_max_) and to collect blood lactate samples at various workloads. The test was conducted following the protocol used by Roig and colleagues [14]. A 5 min warm-up with 75W preceded the graded test on the cycle ergometer (Ergomedic 939E, Monark, Sweden). Subjects were required to maintain a cadence of ≥80RPM during the warm-up and the duration of the test. Immediately following the warm-up resistance was increased to 100W and then increased with increments of 50W every 3 min. Subjects were instructed to cycle to exhaustion and strong verbal encouragement was given during the test.

Pulmonary ventilation, oxygen consumption, heart rate (Polar Electro, Kempele, Finland), exhaled CO_2_ and respiratory exchange ratio (RER) were measured continuously and updated online every 15s (MasterScreen CPX^®^, Carefusion, Germany). Measures of blood lactate were collected and determined with the finger prick method from the non-dominant hand (Accutrend^®^ Plus System, Roche Diagnostics, Switzerland) at rest prior to the test start, during the last 30s of each work interval, at exhaustion and 5 min after exhaustion. VO_2peak_ was determined when at least one of the following criteria was met: a plateau in the VO_2_ curve, an RER ≥ 1.1, an inability to maintain 80RPM and/or volitional exhaustion. Mean values for relative VO_2peak_ and W_max_ for each group can be seen in Table 1.

### Exercise Protocol

The exercise protocol for EX45 and EX90 was similar to that from the studies of Roig et al. [14] and Mang et al. [15]. The total duration of the exercise was limited (17min) in order avoid excessive fatigue and/or dehydration, which could potentially have a negative effect on memory processing [31, 32]. Subjects were required to complete a four minute warm-up, after which they completed 3 intervals of 3min duration on a cycle ergometer separated by a 2min active rest interval while keeping a cadence of ≥80RPM. Heart rate was monitored and recorded (Polar Electro, Kempele, Finland) for the duration of the exercise bout, rate of perceived exertion (RPE) values (Borg Scale) [33] were recorded during work and active rest intervals and blood lactate measurements were taken at rest prior to exercise, at completion of each work interval then again at 5 min post exercise completion.

The work and active rest intensities for the two exercise groups were calculated on the basis of the maximum power output (W_max_) achieved during the graded maximal exercise test. For the high intensity group, EX90, the protocol was designed to ensure high levels of blood lactate (≥10mmol/L) and was performed in the following way; 2min at 100W, 2min at 60% W_max_, 3min at 90% W_max_, 2min at 60% W_max_, 3min at 90% W_max_, 2min at 60% W_max_, 3min at 90% W_max_. The exercise format for the EX45 group was the same with the work load adjusted during the warm-up to 50W and in the three 3 min intervals set at 45% of W_max_ and the rest intervals at 25% of W_max_.

### Study Design

**Fig 2:**
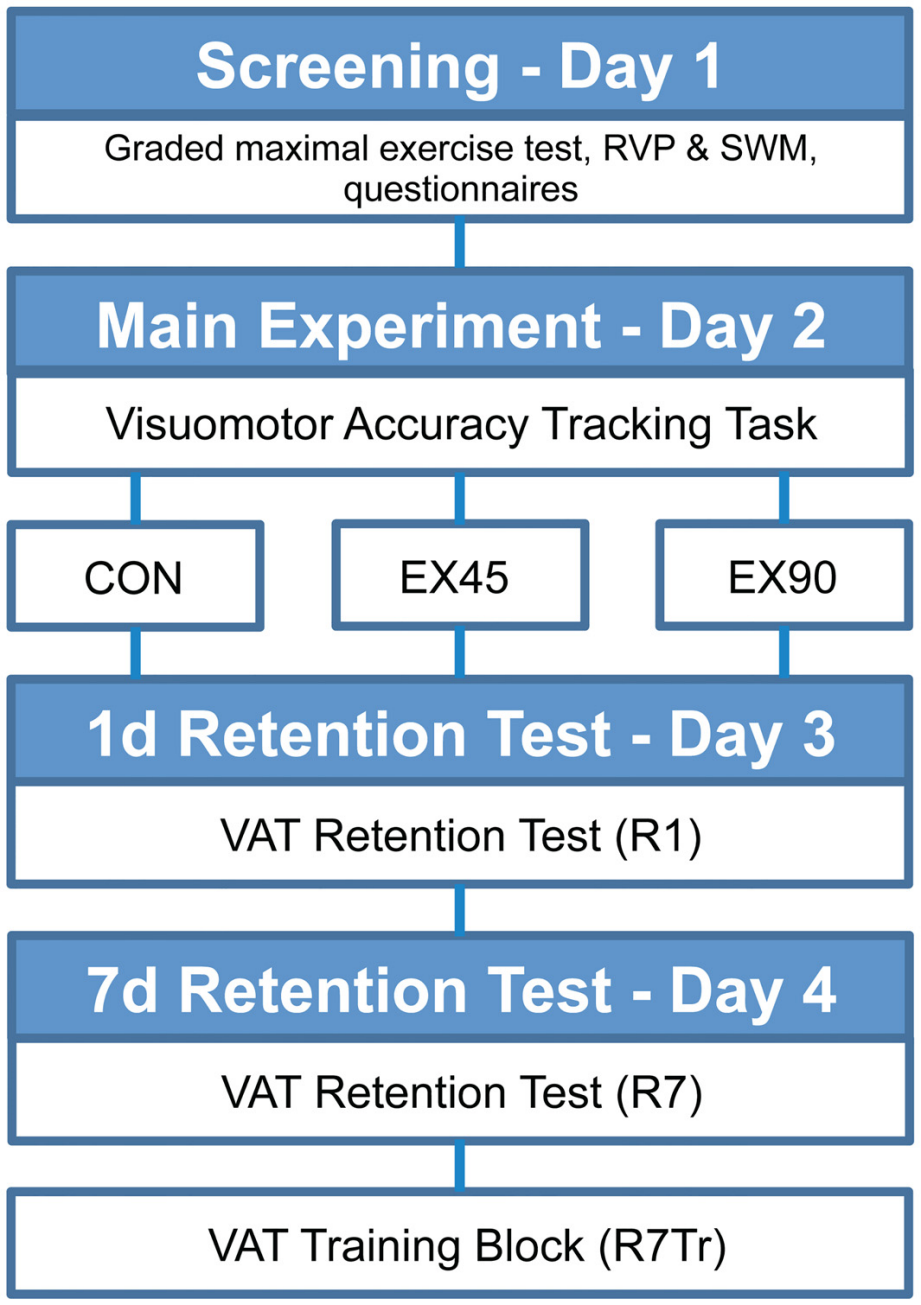
Study Design. Schematic illustration of the study design.

A schematic illustration of the study design can be seen in Fig 2. Subjects were required to visit the laboratory on four separate occasions with the aim of assessing the effects of a low (EX45) and high (EX90) intensity exercise bout respectively on the consolidation of a newly acquired motor skill measured with delayed retention tests. The first visit involved screening for preliminary and baseline measurements and subjects performed the graded maximal exercise test.

At least 1 day after the screening session, subjects returned to the laboratory to complete the main experimental session and were required to refrain from exercising during this period. Subjects then returned to the laboratory exactly 1 day and 7 days after the main experiment to complete the retention tests, R1 and R7 respectively. All sessions were carried out at the same time of day (± 2h). Randomization was stratified to ensure that the groups were matched for age, BMI, relative peak oxygen consumption (VO_2peak_: mlO_2_·min^-1^·kg^-1^) and baseline score in the VAT. The subjects were required to abstain from physical activity 2 hours before and 4 hours after the tests sessions. They were also required to refrain from caffeinated products in the same time frame [34].

### Main Experiment and Retention Tests

On arrival at the laboratory subjects were required to complete the Positive and Negative Affect Schedule scale (PANAS) [35]. The scale was used to determine positive (PA) and negative (NA) affects before VAT acquisition, R1 and R7 respectively. Subjects completed the Stanford Sleepiness questionnaire [36] prior to starting VAT acquisition. Following the completion of block 3 of the VAT subjects completed a flow questionnaire relating to how they evaluated their mental state and performance [37]. This was the Danish version of the 13-item Flow Kurz Skala [38] which has been previously used and described by this group [39]. Similarly an Intrinsic Motivation Inventory (IMI) [40] was filled out on completion of the VAT.

At 20min post VAT the subjects assigned to the control group (CON) remained seated while the two exercise groups completed the standardized acute exercise bout on a cycle ergometer in an adjacent laboratory. The main experiment was concluded with the completion of the Montréal Sleep Diary for the night preceding the main experiment. This was an adapted sleep questionnaire based on the Pittsburgh Sleep Diary [41], which has been used in numerous studies [42–44].

At the 1 day retention test (R1) subjects were required to complete a retention test in the VAT, without feedback. The removal of feedback (performance score) at the retention tests was done to exclude any learning effects, which might relate to receiving feedback such as guidance and motivation [30]. This format was also repeated at the 7 day retention test (R7) and an additional training block (R7Tr) in the VAT with feedback was completed in order to check for continued learning potential and thus rule out a potential ceiling effect in skill improvement. Both retention tests were concluded with the Montréal Sleep Diary.

### Neuropsychological Tests and Questionnaires

On Day 1 (Screening), all subjects performed standardized neuropsychological tests of spatial working memory (SWM) and sustained attention (rapid visual processing, RVP) with CANTAB software (Cambridge Cognition Ltd, UK). Questionnaires were completed including the Achievement Motives Scale (AMS) [45], the Task and Ego Orientation in Sport Questionnaire (TEOSQ) [46], the International Physical Activity Questionnaire—Long (IPAQ) [47], flow proneness [39, 48] and health background via a standardized general eligibility questionnaire.

### Statistical Analysis

VAT parameters, exercise parameters and parameters from tests of sustained attention, spatial working memory, PANAS, sleepiness, physical activity level and sleep were all analyzed by means of linear mixed models with group-time interactions as fixed effects and subject-specific random effects were fitted. Additionally, subject-by-time random effects were also included in the model for VAT score to account for repeated measurements from the trials. The random effects captured inherent variability between subjects. Separate models were fitted for the VAT acquisition phase and the retention tests R1 & R7. Model checking was based on residual plots and normal probability plots using the raw residuals.

The primary hypotheses addressed in this study corresponded to testing specific differences in changes across the time points B5, R1, and R7, specified in terms of contrasts of parameter estimates of the interaction effect (e.g., [49]). Specifically, approximate global F-tests were carried out and, subsequently, model-based t-tests were used to identify the significant differences. The resulting p-values were obtained from a standard normal distribution and multiplicity adjusted using the single-step method in order to control inflation of family-wise type I error rate.

Additionally, changes within intervention groups as well as between-group differences were compared pairwise using model-based t-tests. As these comparisons were exploratory no adjustment of p-values was applied. Data are reported as mean ± SEM unless otherwise stated; where appropriate data are reported with 95% C.I. A significance level of 0.05 was applied.

All statistical tests were carried out in R (R Core Team, 2015). A linear mixed model approach was applied to the data using the functionality of the packages *lme4* [50], whereas the specific comparisons and the corresponding t-tests and adjusted p-values were calculated using the package *multcomp* [51].

## Results

### Subject Information

All subjects were in similar affective status (PANAS) as can be seen from the values in Table 2. No differences were observed between groups for RVP, SWM and IPAQ levels at baseline (Table 2).

**Table 2:** Subject Data.

	CON	EX45	EX90
RVP (Total Hits)	21.0 ± 1.0	20.3 ± 1.4	23.1 ± 1.1
SWM (Total Errors)	11.6 ± 3.8	13.0 ± 2.6	11.9 ± 2.6
PANAS (PAS)			
-Main Experiment	30.3 ± 7.6	30.6 ± 3.3	28.7 ± 6.3
-R1	28.9 ± 8.1	31.0 ± 6.1	28.9 ± 7.7
-R7	28.2 ± 9.7	29.1 ± 6.2	28.6 ± 9.1
PANAS (NAS)			
-Main Experiment	11.8 ± 2.1	12.8 ± 2.5	12.3 ± 1.8
-R1	11.7 ± 1.9	11.6 ± 1.9	11.8 ± 1.8
-R7	11.9 ± 3.6	10.9 ± 1.9	10.8 ± 0.9
Stanford Sleepiness	2.6 ± 0.5	2.7 ± 0.7	2.8 ± 0.9
Montréal Sleep Diary (h)			
-Main Experiment	7.8 ± 1.1	6.9 ± 1.1	7.3 ± 1.4
-R1	7.6 ± 1.1	8.1 ± 1.2*	7.6 ± 1.0
-R7	7.6 ± 0.8	7.8 ± 1.3*	7.4 ± 1.0

Subject data for tests of sustained attention, spatial working memory, PANAS, sleepiness, physical activity level and sleep (group mean ± SD).

* Significant difference from value at Main Experiment.

RVP = Rapid Visual Processing, SWM = Spatial Working Memory, PANAS = Positive and Negative Affect Schedule

### Physiological Response

**Fig 3:**
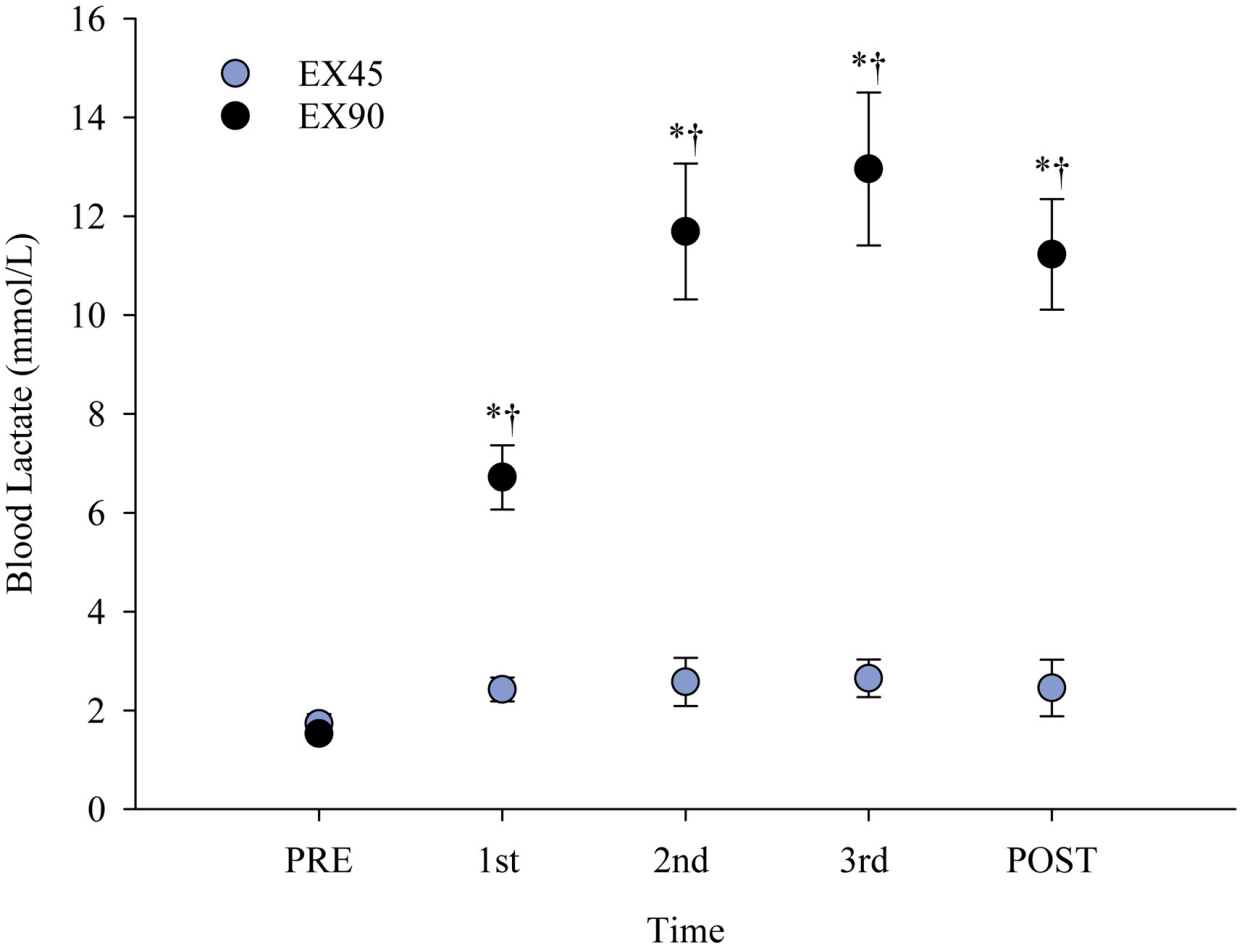
Blood lactate levels. Mean blood lactate levels (mmol/L) for the low (EX45) and high (EX90) intensity exercise groups (± SEM) at time points PRE, interval 1, 2 & 3 and 5 min POST exercise. * Significant difference compared to PRE values. † Significant inter-group difference at time point (p<0.05).

Group mean values for the low and high intensity bouts are presented in Table 3. A significant difference between groups was observed for every exercise parameter (all p≤0.05) apart from baseline blood lactate levels. Blood lactate levels for time points pre, interval 1, 2 & 3 and 5 min post exercise are presented in Fig 3. There was a significant Group x Time interaction between EX45 and EX90 for blood lactate levels (*F*_4,102_ = 30.01 p<0.001), heart rate (*F*_11,130_ = 128.10 p<0.001) and RPE (*F*_11,130_ = 50.62 p<0.001).

**Table 3:** Exercise Data.

	EX45	EX90
(Work) Watt (W) 45/90% W_max_	146.5 ± 18.0	285.0 ± 39.9*
(Active Rest) Watt (W) 25/45% W_max_	81.5 ± 10.0	190.0 ± 26.6*
Baseline Lactate (mmol/l)	1.7 ± 0.7	1.5 ± 0.4
Peak Lactate (mmol/l)	2.7 ± 1.3	13.0 ± 5.4*
(Work)RPE	12.7 ± 1.1	17.0 ± 1.8*
(Active Rest)RPE	10.5 ± 1.5	13.7 ± 2.2*
(Work) Heart Rate (beats/min)	132.5 ± 14.7	173.6 ± 13.2*
(Active Rest) Heart Rate (beats/min)	113.2 ± 13.5	152.1 ± 13.6*

Exercise data for EX45 & EX90 groups (mean values ± SD).

RPE = Rating of Perceived Exertion

* Significant between-group difference (all p≤0.05)

### VAT Acquisition

**Fig 4:**
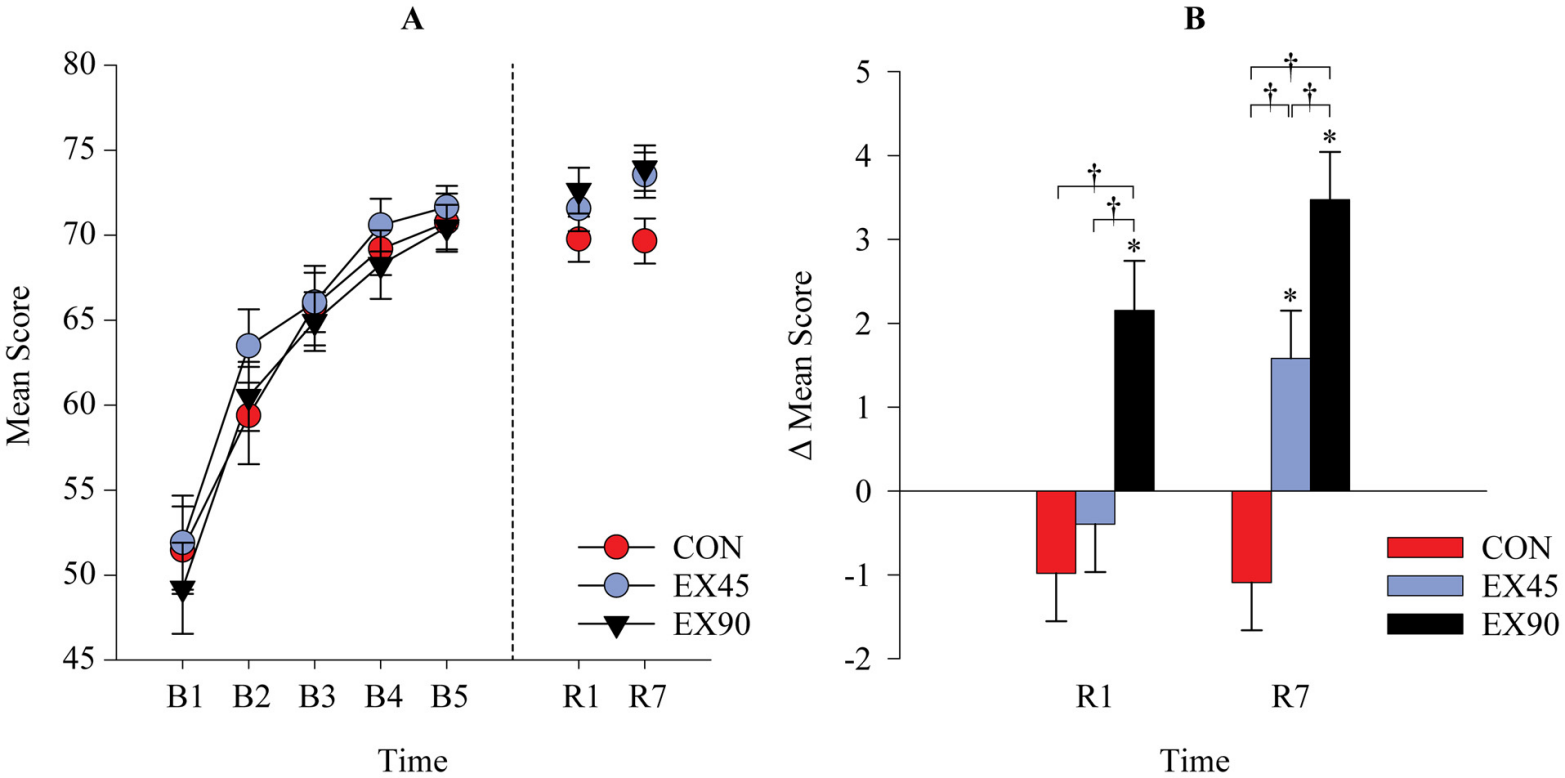
Performance scores in the visuomotor accuracy tracking task (VAT). **A**: Mean scores (± SEM) in the VAT at acquisition Baseline (B1), B2-4 and B5. **B:** Changes in mean scores (± SEM) for all groups in the VAT from Block 5 (B5) to 1d retention test (R1) and 7d retention test (R7). * Significant change from B5 (p = 0.004). † Significant between-group difference (p<0.05).

From Baseline (B1) to B5 all groups showed similar significant improvement (*F*_*14*,*3399*_ = 155.1 p<0.001), with a mean increase of 45.65 ± 0.04% equivalent to an increase of 21.02 ± 1.04 in mean score (Fig 4). There were no between-group differences at Baseline (B1), or at B2, B3, B4 or B5. This indicates that skill improvements during acquisition and, more importantly, skill level at the end of acquisition, were similar among groups (Fig 4A).

### VAT Retention Tests

There was a significant Group x Time interaction between B5-R1 (*F*_4,2020_ = 9.35 p<0.001). Changes in mean scores from B5 to R1 for EX90 were greater than CON 3.14 ± 0.82 (p<0.001) and these changes were also greater than the EX45 group 2.55 ± 0.82 (p = 0.011). A significant Group x Time interaction was observed between B5-R7 (*F*_2,2020_ = 16.18 p<0.001). The EX90 group experienced greater changes in score 4.56 ± 0.81 (p<0.001) from B5 to R7 compared to the CON group. Furthermore, there was a significant difference of 1.89 ± 0.81 (p = 0.049) between the EX90 group compared to the EX45 group from B5 to R7 and similarly a significant difference of 2.67 ± 0.81 (p = 0.003) between the EX45 group compared to the CON group between the same time points (Fig 4B).

There was a significant Group x Time interaction between mean values at B5-R1-R7 (*F*_8,2020_ = 7.22 p<0.001). For the EX90 group there was a significant increase of 3.47 ± 0.57 (p<0.001) in mean score at R7 compared to B5 (Fig 4A) corresponding to a relative increase of 5.12 ± 2.02% (B5 70.48 ± 1.33; R7 73.95 ± 1.33). There was also a significant increase of 2.15 ± 0.59 (p<0.001) in mean score at R1 compared to B5 and additionally from R1 to R7 1.32 ± 0.59 (p = 0.025) (B5 70.48 ± 1.33; R24 72.63 ± 1.34; R7 73.95 ± 1.33). Likewise, for the EX45 group there was a significant increase of 1.58 ± 0.57 (p = 0.006) in mean score at R7 compared to B5 (B5 71.96 ± 1.33; R7 73.54 ± 1.33), a relative increase of 2.24 ± 0.85%. There was also a significant increase of 1.98 ± 0.57 (p<0.001) from R1 to R7 for the EX45 group (R24 71.56 ± 1.33; R7 73.54 ± 1.33). Mean changes in scores for the CON group from B5 to R1 were -0.98 ± 0.57 (p = 0.085) and at R7–1.09 ± 0.57 (p = 0.056) but these did not reach statistical significance.

At R7 a significant between-group difference in mean scores of 4.29 ± 1.89 (p = 0.023) was observed between CON and EX90 (CON69.66 ± 1.33; EX9073.95 ± 1.33). Likewise, at R7 the difference in mean scores between CON and EX45 was 3.89 ± 1.89 (p = 0.040; CON 69.66 ± 1.33; EX45 73.54 ± 1.33).

### 7 Day Retention (R7) Training Block R7Tr Comparison

The Group x Time interaction between mean values at R7-R7Tr was significant (*F*_2,1359_ = 3.71 p<0.025). Changes in performance score from R7 to R7Tr for CON were greater than EX90 2.11 ± 0.55 (p<0.001). There was a significant difference in mean performance score between time points R7 and R7Tr for CON 2.32 ± 0.58 (p<0.001) (R7 69.66 ± 1.34; R7Tr 71.98 ± 1.34) and EX45 1.18 ± 0.55 (p = 0.031) (R7 73.54 ± 1.34; R7Tr 74.72 ± 1.34). Values for EX90 were R7 73.95 ± 1.34: R7Tr 74.17 ± 1.34 (p = 0.691).

## Discussion

The hypothesis for this study was that there exists a dose-response relationship between the intensity of an acute aerobic exercise bout and the corresponding level of retention in a visuomotor tracking task measured as changes in performance scores 1 and 7 days after motor practice. Our main result supports this hypothesis demonstrating that a significantly greater change in mean scores was observed for the high intensity exercise group (EX90) at 1 and 7 days compared to both the low intensity (EX45) and resting control (CON) groups. The CON group demonstrated a tendency towards an off-line decrease at 24h with a further decline at 7 days. While the values for CON did not reach statistically significant levels, the between-group differences between the control group and the EX90 and EX45 groups were significant at the 7 day retention test. Following the 7 day retention test only the CON and EX45 groups improved motor performance with continued practice. This could indicate that EX90 had significantly larger offline gains in motor performance compared to CON and EX90 and were approaching a possible ceiling effect in the VAT task, and that the task may, to some extent, have limited the effects observed for EX90.

We provide here additional information regarding the influence of one of the several parameters within exercise, which can potentially affect the consolidation of procedural memory. Our results suggest that intensity of the acute exercise bout following motor skill acquisition is central to stimulating or amplifying the cellular mechanisms in the central nervous system that underlie the consolidation process. There appears to be a connection between exercise intensity, consolidation and the retention of procedural memories. It is well established that systemic blood flow increases in line with a concurrent increase in exercise intensity and these are accompanied by increases in biomarkers [3, 16, 52]. Similarly, a larger percentage of skeletal muscle must be recruited as intensity increases involving a greater contribution from the central nervous system. Whether exercise has an effect at a systemic level increasing blood flow and activity in the central nervous system, or whether it is one or more of the biomarkers released during exercise that contribute to this amplification effect is uncertain. It is possible that it is a combination of multiple factors, which the following section will address.

Based on the results of Skriver et al. [16] lactate appears to be an important biomarker of the effect of exercise on motor memory. It does not necessarily indicate that it is an elevated blood lactate level *per se*, which triggers amplification of the plastic changes occurring at the synaptic level. The increased production and release of lactate during high intensity exercise potentially represents a signaling mechanism or mediator compound relating to the release of BDNF [53] and motor memory formation [16, 54]. It is however unclear how the interplay and contributions of lactate and various different compounds in humans affect learning and how exercise seems to amplify these effects. Exercise at higher relative intensities have shown to lead to greater release of a number of biomarkers of which BDNF and norepinephrine (NE) correlate with an increased level of retention of a motor skill [16] although associations between the peripheral concentration of some of these neurochemicals and procedural memory are not supported by all studies [15]. Other compounds, which may also play a role in memory processing, namely epinephrine, dopamine and insulin like growth factor 1 (IGF-1) [16] are possible contributors having modulating effects. Peripheral measures of these compounds and their role in plastic changes within the central nervous system must be approached with caution as they are not necessarily an accurate correlate or predictor of improvements in motor skill learning [16].

Similar to the 2012 study by Roig and colleagues [14] we observed a more pronounced difference in retention level after 7 days. This may be related to the proposed phases of memory consolidation presented by Dudai and co-workers [55, 56]. Newly acquired memories, in this case motor memory, are consolidated first of all at the cellular/synaptic level over a period of minutes and hours following encoding and termed synaptic consolidation. The post-encoding reorganization of long-term memory lasting days and years involving structural and network changes is termed as systems consolidation and could partly explain the continued increase in retention of the motor skill at 7 days. There is evidence to suggest that exercise may protect newly established memory traces. This has been demonstrated by the recent study by Rhee and co-workers [22]. While the type of task differed from the visuomotor accuracy tracking task used here, exercise, placed in close temporal proximity to the encoding phase of a motor skill, would seem to augment the consolidation process [23, 57]. The focus of the Rhee study was on the effect of exercise in relation to interference and a broad enhancement and protection of the off-line gains of task A was observed when performed immediately prior to a second interfering task (B) at 2h post.

During the consolidation phase procedural memory can potentially be enhanced and/or stabilized [55, 56, 58, 59]. There is also the potential for interference during this phase [60–63] but it would seem that the placement of exercise here can lead to an enhancement of the memory [14]. This further underlines the extent to which consolidation is susceptible to both positive and negative influences. The acquisition and retention of motor skills have been shown to be susceptible to changes in and/or states of awareness [64], sleepiness, mood [48], motivation and relating to motivation the type and timing of feedback and/or cues [17, 64]. The homogeneity of the three groups at the outset of the study where subjects were also matched for age, BMI, relative aerobic capacity and baseline score in the visuomotor tracking task (Tables 1 & 2), allows us to more confidently attribute the differences in retention between groups to be related to the exercise intervention.

Regarding the potential for incorporating exercise into motor skill learning scenarios there are a number of considerations. It would seem that an acute bout of high intensity aerobic exercise following acquisition of a new motor skill improves the skill level by promoting an increased level of long-term retention. This may be a viable method with healthy, moderately trained individuals, as shown in this study, but may not be a viable alternative for certain patient groups in a rehabilitation setting or the elderly [65]. Performing a bout of high intensity exercise is both physically and mentally taxing and if a similar positive effect on retention could be elicited by reducing the intensity then this would provide a more practically viable option [66].

The effects of low intensity exercise on retention seem to be present but not as pronounced as the effects of high intensity exercise. The work conducted by Statton and co-workers [20] and Snow et al. [21] on moderate intensity exercise and skill acquisition, therefore, can be seen to support this finding although it is important to underline the fact that the exercise bout was placed prior to motor skill acquisition in these studies. In the present study exercise was placed *following* skill acquisition and low intensity exercise led to a significant change in mean score for the low intensity group compared to the control group after 7 days. The larger positive effects observed for intense exercise do not necessarily mean that moderate or low intensity exercise cannot be applied, but rather that it is a trade off in certain groups between improvements in motor skill learning and responsible, viable practices in a clinical setting. Studies applying exercise in a clinical setting for the sick, weak or elderly will provide additional information [67]. The practical application of the results from this study in a clinical setting must also be approached with caution as the participants here were young healthy males with relatively high aerobic fitness levels. While high intensity exercise could be applied in a clinical setting or for the elderly it would be necessary to screen for contra indications and take appropriate precautions for safety, whereas low intensity exercise bouts provide a viable alternative.

## Conclusions

An acute bout of high intensity aerobic exercise improves consolidation of a visuomotor tracking task (VAT) where significant improvements are seen at 1 and 7 days post acquisition of the motor skill. These improvements were observed both in the low and high intensity exercise groups compared to a control group that rested post acquisition. Importantly, the high intensity exercise group demonstrated a higher level of retention than both the control and low intensity group at 1 and 7 days. This result suggests that there is a dose-response relationship between exercise intensity and retention level in this type of task when exercise is performed 20 min after motor skill acquisition. The results also emphasize the importance of studying the effects of exercise intensity in modulating potential mechanisms underlying the effects of acute exercise on motor memory.
